# Towards Tobacco-Free Generation: implementation of preventive tobacco policies in the Nordic countries

**DOI:** 10.1177/14034948221106867

**Published:** 2022-07-07

**Authors:** Anu Linnansaari, Hanna Ollila, Charlotta Pisinger, Janne Scheffels, Jaana M. Kinnunen, Arja Rimpelä

**Affiliations:** 1 Faculty of Social Sciences, Unit of Health Sciences, Tampere, Finland; 2 Department of Public Health and Welfare, Finnish Institute for Health and Welfare, Helsinki, Finland; 3 Center for Clinical Research and Prevention, Bispebjerg and Frederiksberg Hospital, The Capital Region of Denmark, Denmark; 4 Faculty of Health Sciences, University of Copenhagen, Copenhagen, Denmark; 5 Danish Heart Foundation, Copenhagen, Denmark; 6 Department of Alcohol, Tobacco and Drugs, Norwegian Institute of Public Health, Oslo, Norway; 7 Department of Adolescent Psychiatry, Tampere University Hospital, Tampere, Finland

**Keywords:** Tobacco policies, tobacco prevention, tobacco control, WHO FCTC, tobacco-free generation, tobacco endgame, policy implementation, policy implementation research, policy adoption, the Nordic countries

## Abstract

**Aims:**

Europe’s Beating Cancer Plan set a goal of creating a Tobacco-Free Generation in Europe by 2040. Prevention is important for achieving this goal. We compare the Nordic countries’ preventive tobacco policies, discuss the possible determinants for similarities and differences in policy implementation, and provide strategies for strengthening tobacco prevention.

**Methods:**

We used the World Health Organization Framework Convention on Tobacco Control (WHO FCTC) to identify the key policies for this narrative review. We focused on Articles 6, 8, 9, 11, 13 and 16 of the WHO FCTC, and assessed the status of the required (core) and recommended (advanced) policies and their application to novel tobacco and nicotine products. Information on the implementation of strategies, acts and regulations were searched from global and national tobacco control databases, websites and scientific articles via PubMed and MEDLINE.

**Results:**

The WHO FCTC and European regulations have ensured that the core policies are mostly in place, but also contributed to the shared deficiencies that are seen especially in the regulations on smokeless tobacco and novel products. Strong national tobacco control actors have facilitated countries to implement some advanced policies – even as the first countries in the world: point-of-sale display bans (Iceland), outdoor smoking bans (Sweden), flavour bans on electronic cigarettes (Finland), plain packaging (Norway), and plain packaging on electronic cigarettes (Denmark).

**Conclusions:**

Collaboration and participation in reinforcing the European regulations, resources for national networking between tobacco control actors, and national regulations to provide protection from the tobacco industry’s interference are needed to strengthen comprehensive implementation of tobacco policies in the Nordic countries.

## Introduction

In 2021, the goal of Tobacco-Free Generation in Europe was set in Europe’s Beating Cancer Plan, with less than 5% of the population using tobacco products by 2040 [1]. Tobacco prevention is key to achieving this goal. Preventive policies are integral in the provisions of the World Health Organization Framework Convention on Tobacco Control (WHO FCTC) [2], which requires that countries implement effective measures and cooperate with others in developing policies for the prevention and reduction of tobacco consumption, nicotine addiction and exposure to tobacco smoke. Comprehensive implementation of the key WHO FCTC policies has been shown to be important in diminishing tobacco use [3].

All the Nordic countries had acceded to the WHO FCTC already by 2005 and have succeeded in reducing adolescent daily smoking, which in 2019 ranged from 1.9% in Iceland to 10% in Denmark. Smokeless tobacco and novel products, such as electronic cigarettes (e-cigarettes) and nicotine pouches, are creating new challenges for prevention. In 2019, e-cigarette use during the last 30 days was most common in Iceland (17%) and least common in Sweden (6.4%) [4]. In a recent Nordic comparison, the proportion of dual and triple users among youths was high [5]. In addition, variation remains in the comprehensiveness of the implementation of tobacco control policies. In the most recent European tobacco control scale from 2019, Iceland was fourth, Norway fifth, and Finland sixth in a comparison of the key tobacco control measures across 36 European countries, with no major changes since 2010. For Sweden, the ranking has declined from 9th to 15th, and for Denmark from 13th to 29th [6, 7].

Despite the need for accelerating the implementation of tobacco policies, research has mainly focused on the impact of policies rather than on their adoption and implementation. Perceiving implementation outcomes as health benefits may partly explain the lack of focus on implementation itself in health policy research [8, 9]. However, differentiating implementation outcomes (i.e. adoption, feasibility and fidelity) from health outcomes makes visible what is required from the implementation to reach the targeted health outcomes [10]. Furthermore, evidence on effectiveness does not solely explain the adoption of policies [11], and therefore understanding complex policymaking processes is crucial to increase the adoption and implementation of tobacco policies [12]. The need for increasingly integrating research on public health, implementation and politics has been debated during the past decade [13–18].

In this study, we integrate insights from these contributing disciplines to understand better and overcome challenges in the adoption and implementation of preventive tobacco policies. We aim to assess and compare the comprehensiveness of preventive tobacco policies in the Nordic countries, focusing on the implementation of the WHO FCTC policies and their application to novel tobacco and nicotine products. In addition, we discuss what determinants may underlie the similarities and differences in the policy implementation and consider how countries have impacted each other. While we acknowledge that the practical implementation and enforcement of the national legislation is an essential parallel process, in this study we consider the policies implemented when the respective provisions in national laws or other regulations have been enacted. Our objective is to provide strategies for strengthening the comprehensive implementation of tobacco policies to target the Tobacco-Free Generation at the national and Nordic levels. The research questions for the study are:

1. How comprehensively are the preventive WHO FCTC policies implemented in the Nordic countries? What similarities and differences exist between the countries? How have the Nordic countries influenced each other’s policy adoption and implementation?
2. What national, European and global determinants may have influenced policy adoption and implementation in the Nordic countries? How have they facilitated or hindered the policy adoption and implementation?

## Methods

Our study is a narrative review forming a scholarly summary, along with an interpretation and critique [19], that iteratively follows the stages suggested by Mays et al. [20]. The method provided us with an opportunity to extend the existing understanding on the adoption and implementation of preventive tobacco policies in the Nordic countries by synthesising multifaceted and fragmented information from various sources. Regarding the five stages of producing public policies – agenda setting, policy formulation, adoption/decision-making, implementation and evaluation [21] – we mainly focus on explaining the adoption phase. In line with the implementation outcome frameworks [9, 10], policy ‘adoption’ is defined as our implementation outcome. In this study, the adoption indicates the decision-making process leading (or not) to tobacco policies being implemented at the national level, namely to the enactment of the respective provisions in the national laws and regulations.

We focused on six WHO FCTC provisions that are most relevant in the light of scientific evidence for preventing uptake and exposure to tobacco and nicotine in adolescence: taxation and price policies (Article 6), protection from exposure to environmental tobacco smoke (Article 8), product regulation (Article 9), packaging (Article 11), advertising and promotion (Article 13), and preventing product access by minors (Article 16). Under these, we categorised required measures as core policies, and recommended measures as advanced policies (see Supplemental file 1 for indicators). The WHO FCTC applies by default to all tobacco products: cigarettes, roll-your-own (RYO), pipe tobacco, water pipe tobacco and smokeless tobacco. To the extent possible, we assessed the application of the core and advanced policies also to the following novel and emerging products: e-cigarettes, heated tobacco products (HTPs) and nicotine pouches. The behaviour change wheel (BCW) [22] guided the classification of the policies and helped in selecting a comprehensive set of policies as implementation objectives for the study. The BCW is an evidence-based framework that summarises policy and intervention measures that influence behaviour through motivation, capability and opportunities [22].

An initial search for information on the strategies, acts and other regulations in Denmark, Finland, Iceland, Norway and Sweden was performed by a research assistant, who compiled relevant information from the latest national tobacco control strategy documents that were identified in the national languages from governmental online databases and the websites of these countries, as well as the websites of non-industry-affiliated and non-governmental organizations (NGOs). Scientific articles and news were searched to identify information on policy changes, after which the search was broadened to the WHO FCTC implementation database [23] and the World Health Organization (WHO) global tobacco control policy data [24]. Based on the information obtained from these sources, the research assistant compiled a narrative describing the current regulatory scheme in each country and a summary of key differences and similarities. This was reviewed by the project team to identify the need for further information and validation. The research assistant then gathered additional information and amended the narrative with support from three researchers. The updated summary of policy comparisons (RQ1) was then sent for feedback to the health authorities in the Nordic countries in December 2020.

**Figure 1.**
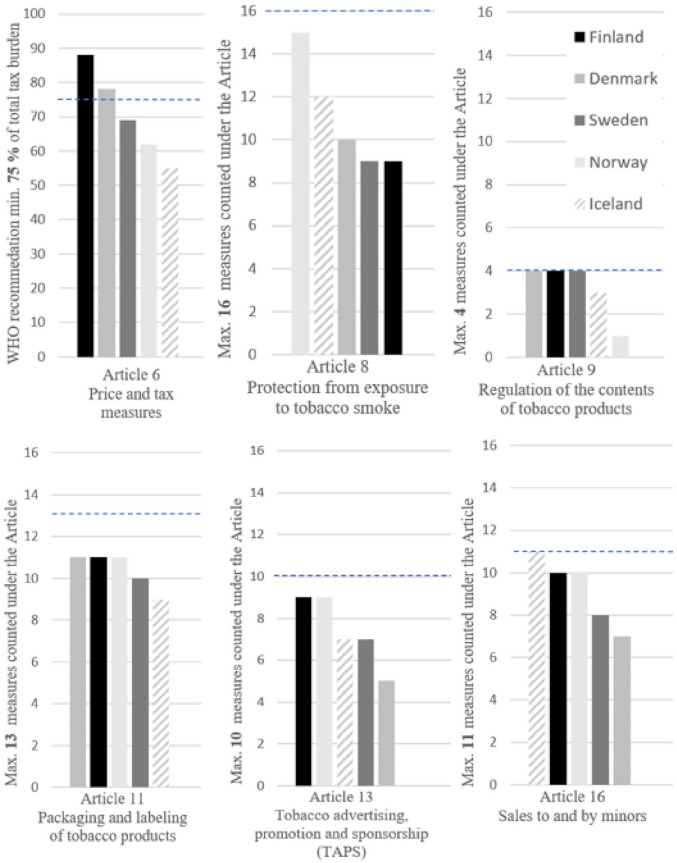
Comparisons of the comprehensiveness of the core and advanced preventive WHO FCTC policies in the Nordic countries. The horizontal dashed line indicates the maximum number of measures counted under the article (Articles 8, 9, 11, 13 and 16) or the recommended minimum level of implementation (Article 6). The included measures are described in Supplement 1.

After the health authorities’ feedback, the results on policy comparisons were revised by two researchers in close collaboration with the project team. This process included screening again the initial data sources and searching for additional data on strategies, acts and other regulations from the WHO FCTC implementation database [23], the WHO global tobacco control policy data [24] and governmental and NGO online databases and websites. To maintain the readability of the results section, all referenced databases, acts and regulations have been compiled and presented in Supplemental file 2 as part of the detailed policy comparisons of the selected articles. Initially, Article 15 (illicit trade) was included in the search topics, but it was removed at this stage after a decision to focus the paper on youth and prevention. To provide comparable estimates of the comprehensiveness of implementation of the WHO FCTC measures, we utilised publicly available data from the WHO FCTC implementation database [23], and one researcher counted the number of implemented policies or measures under the selected provisions for every country (see Figure 1). For Article 8, both complete and partial bans were counted. Tax measures in Article 6 are derived from the 2021 WHO global tobacco control policy data [24].

The research questions were amended with the project team also to include the policy adoption and implementation aspect (RQ2). Our initial understanding of the main national, Nordic, European and global determinants for policy adoption and implementation was guided by the WHO FCTC and the core determinants for policy change: societal factors, institutions, networks/interest groups, agenda setting/framing and ideas [25] (Supplemental file 1). To analyse further how these factors may have influenced tobacco policy adoption and implementation in the Nordic countries, two researchers searched for grey literature via governmental and NGO websites (language: all national languages, timeline: 1990–2021) and key scientific articles via PubMed and MEDLINE (language: English, timeline: 1990–2021, keywords: tobacco polic*, preventive tobacco polic*, WHO FCTC, adopt*, implement*). Data were selected for further analysis if they provided information on the possible determinants and processes for tobacco policy adoption and implementation in the Nordic countries. We also used snowballing techniques to identify clusters of evidence. After the analysis was finished and the whole research team agreed with the results, the health authorities were further contacted for feedback on the results of RQ1 (November 2021) and for the results of RQ2 and revised results of RQ1 (January 2022). The feedback concerned validation of the information and led to only minor changes. The interpretation of the information is the sole responsibility of the authors.

## Results

### 1. Preventive tobacco policies in the Nordic countries

#### 1.1. Core policies

All the core policies required by the WHO FCTC were implemented at least to some extent in all countries. These include the 18-year age limit for sales, comprehensive prohibitions on tobacco use in schools, smoking bans in indoor public places, comprehensive bans on tobacco advertising, promotion and sponsorship (TAPS), and warning labels on cigarette packages. Iceland (implemented in 1969–1971, 1985) and Norway (implemented in 1975) were among the first in the world to require health warnings. Norway has the strictest indoor smoking bans, whereas the most exceptions to the bans are found in Denmark. Denmark implemented age control measures later than the other countries; tobacco sales to minors were prohibited in 2004 with an age limit of 16 years, which was raised to 18 years in 2008.

All the Nordic countries have implemented tax policies on tobacco products, but by 2021 only Finland and Denmark had met the WHO recommendation of a minimum of 75% tax share of the retail price of tobacco. In recent years, Denmark has increased tax considerably, whereas Finland has increased taxes in small steps regularly since 2009. Both countries are now among the countries with the highest total tax in the European Union (EU). In Finland, Sweden and Norway, cigarettes became less affordable between 2010 and 2018, and they became less affordable in all Nordic countries from 2018 to 2020. Between 2018 and 2020, the change was the highest in Denmark and Finland and the smallest in Sweden and Iceland.

Sweden, Iceland and Denmark do not report that the advertising ban in TAPS also covers the global Internet. However, the WHO FCTC implementation guidelines for Article 13 do not provide a clear definition of the global Internet, which may have led to different interpretations of the TAPS bans in this context. Regarding the most recent WHO report [24], direct advertising on the Internet is banned in all Nordic countries. Challenges may arise from indirect advertising and the enforcement of the bans, for instance on social media platforms and advertising with cross-border effects. Iceland and Denmark have not prohibited cross-border advertising originating from their country, nor Iceland cross-border advertising entering the country. Finland is the only country reporting the imposition of penalties for cross-border advertising.

All the WHO FCTC provisions cover smokeless tobacco by default. Snus is sold in Sweden and Norway, chewing tobacco and nasal tobacco in Denmark and nasal tobacco that is also used orally in Iceland. In Finland, selling smokeless tobacco products for oral and nasal use is prohibited, but limited personal imports are allowed. Most of the core policies have been applied to smokeless tobacco products in countries that have smokeless products on the market. Smokeless tobacco is subject to taxation in Iceland (with a total tax of 62%), Norway (64%), Sweden (total tax rate not available) and Denmark (total tax rate not available). Smoking bans are generally not extended to the use of smokeless tobacco, except in schools. Only Swedish legislation does not prohibit the use of snus in schools and on school grounds. In Finland, snus is included in the latest amendment that extended outdoor smoking bans to public playgrounds.

### 1.2. Advanced policies

Many Nordic countries were globally among the first to implement the more advanced measures recommended by the WHO FCTC or its implementation guidelines. Implementation of these advanced policies has extended since, but consistent implementation across the Nordic countries is still lacking. Iceland was the first country in the world to enact a point-of-sale display ban for tobacco products in 2001. All other Nordic countries except Sweden have since implemented the policy. In Denmark, Norway and Iceland, who allow online sales, the display ban also covers online stores, meaning that images of products may not be shown to the customer. In Denmark, images of pipes are excluded from the ban. Finland is the only Nordic country prohibiting the purchase of all tobacco products via distance communication, such as the Internet or email.

Bans on flavours for cigarettes and RYO have been implemented in all Nordic countries except in Sweden and Iceland. Such a ban is, however, also expected in these countries in 2022 as part of their implementation of the EU Tobacco Products Directive (TPD). Prohibitions on flavours in smokeless products have not been implemented in any country. Denmark has introduced a ban on characterising flavours other than tobacco and menthol in other tobacco products than cigarettes and RYO; for example, chewing tobacco (although pipe tobacco and cigars are excepted), but the ban will come into force when the EU law stemming from the TPD allows this. Pictorial warnings were first implemented in Iceland in 1985. None of the countries where smokeless tobacco is sold require pictorial warnings for these products. Norway will also introduce an additional health warning on oral tobacco relating to harms to the fetus in 2022. Norway was the first Nordic country to enact plain packaging in 2017. In Norway, it covers cigarettes, RYO and snus. In Finland, all tobacco products, and in Denmark, all tobacco products except cigars and pipe tobacco will be required to be in plain packaging.

Sweden has been most progressive in extending smoking bans in outdoor or quasi-outdoor public places, including areas outside childcare facilities, public playgrounds, terraces of cafés and restaurants, outdoor areas of public transport, such as bus stops and train stations and entrances to establishments, public venues and other spaces to which the public has access. Also, Norway prohibits smoking outside the entrance to health institutions and public buildings. Finland has implemented smoking bans in audience areas at outdoor public events and recently extended the ban also to playgrounds and public beaches. Tobacco and nicotine-free school hours have been implemented in Denmark and Norway, and also other countries extend the bans to the outdoor premises of schools.

### 1.3. Comprehensiveness of the implemented core and advanced policies

When comparing the countries based on the number of reported core and advanced measures by early 2020 (see Figure 1), the implementation could be more comprehensive in all countries regarding most of the policies. Regulation on the content (Article 9) of tobacco products was rather comprehensive and consistent in the Nordic countries. Country differences can be seen especially in the comprehensiveness of smoking bans (Article 8), taxation (Article 6) and TAPS (Article 13). None of the countries stand out with clearly more comprehensive implementation of the provisions than others, but the countries have different strengths and deficiencies.

In Norway, many policies, such as the smoking ban (Article 8), have been comprehensively implemented, yet the level of taxes (Article 6) and regulations on the content of products (Article 9) could be reinforced. Finland has high taxes (Article 6) and comprehensive prohibitions on TAPS (Article 13), yet the smoking bans (Article 8) could be further strengthened by implementing complete bans instead of partial bans. In Sweden, many policies lack comprehensiveness, yet regulation on the contents (Article 9) is exhaustive – for smoking tobacco. A comprehensive ban on sales to minors (Article 16) has been implemented in Iceland, yet the taxes (Article 6) are low and the regulations on warnings and labelling (Article 11) are less comprehensive than in other countries.

### 1.4. Extending policies to novel tobacco and nicotine products

All the Nordic countries have enacted most of the core policies on e-cigarettes. Overall, Norway has been most strict by banning nicotine-containing e-cigarettes and e-liquids (as well as nicotine pouches) from entering the domestic market; however, after harmonising legislation with TPD, nicotine-containing e-cigarettes will be allowed in 2022. Iceland was the last to adopt the first national legislation on e-cigarettes, doing so only in 2018, with the legislation entering into force in 2019. All countries require a health warning on e-cigarettes and have generally banned vaping in the same indoor areas as smoking. Prohibition on TAPS is fully extended to e-cigarettes in all countries – except Sweden – covering all the same direct and indirect forms of TAPS as for tobacco products, and partially also in Sweden. Finland, Sweden and Norway have excise tax for nicotine-containing liquids and nicotine-free liquids intended for vaporisation. Denmark will introduce a tax on nicotine-containing e-liquids in 2022.

Of the recommended advanced measures applied to e-cigarettes, display bans and flavour bans have received most attention in the Nordic countries. Finland, Norway and Denmark have also applied display bans to e-cigarettes. Finland was the first to prohibit flavours (except tobacco flavour) in e-cigarettes and e-liquids intended for vaporisation. Denmark has banned flavours other than tobacco or menthol in e-cigarettes and e-liquids. Also, the Norwegian, Icelandic and Swedish governments have recently proposed flavour bans in e-cigarettes. Denmark and Finland will require plain packaging for e-cigarettes and refill containers. In Norway, a proposal to extend plain packaging also to e-cigarettes was sent for public consultation in 2021.

The sale of snus-like nicotine pouches is unregulated in Iceland and Sweden. So far, all applications to market nicotine pouches in Norway have been rejected. Finland requires a medical sales permit. In Denmark, nicotine pouches, which are defined as tobacco surrogates, are subject to prohibition on sales to those under 18 years, a display ban, health warnings and restrictions on TAPS, but not a ban on flavours or plain packaging. Furthermore, the Danish parliament has just agreed to introduce a tax on nicotine products such as nicotine pouches from 1 July 2022. The Icelandic government has recently presented a legislative proposal to put nicotine pouches and other nicotine products under legislation comparable to e-cigarettes. It also suggests prohibiting nicotine products, such as nicotine pouches, with appealing flavours; if the legislation is passed, it will be the first of the Nordic countries to do so. In Sweden, government has recently proposed stricter regulations on tobacco-free nicotine products, such as 18-years age limit for sales and strengthening TAPS.

HTPs are sold in Denmark and Sweden, with the total tax being 43% in Sweden and 31% in Denmark. In Denmark, all novel tobacco products, such as HTPs, are subjected to an 18 years age limit for sales, a display ban, a ban on direct and indirect advertising and sponsorship in stores and online and a health warning. Denmark will require plain packaging also on tobacco for HTPs. In Sweden, HTPs are covered by health warnings, and the products are also subjected to the 18 years age limit for sales and regulations on TAPS. Although HTPs are not sold in Finland, the products are already subject to similar regulation as other tobacco products, such as the age limit for sales, smoking bans and taxation. A display ban concerning HTP devices and plain packaging are included in the latest legislative proposal.

### 1.5. Impact of Nordic countries on each other

As the Nordic countries have their individual strengths and deficiencies in policy implementation, it provides a great basis for knowledge sharing and policy diffusion from one country to another. In Finland and Norway, where the display ban was adopted in 2010, the legislative proposals referenced Iceland as one of the countries having already implemented the ban [26, 27]. Iceland implemented the ban in 2001 as the first country in the world (Supplemental file 2). Demonstrating other countries’ more advanced policies may also put pressure on decision-makers to move forward. This was the case in Denmark, where the recent significant improvements in tobacco control were demanded of decision-makers by highlighting the examples of the other Nordic countries [28].

On the other hand, more lenient tobacco policies in some countries may undermine tobacco prevention in other countries. For instance, some countries have a wider selection of tobacco and nicotine products on their national markets, which challenges the strict demand and supply reduction measures in other countries. For example, as Sweden has the exemption to snus sales and it does not regulate traveller exports of snus, it is common to import it to Norway, Finland and Denmark. The incentive to import snus arises both from the national sales ban (in Finland and Denmark) and from lower prices, both compared to cigarettes and local smokeless tobacco [29]. The sale of snus is allowed in Norway, but lower prices have made private imports from Sweden in particular – as well as tax free purchases [30] – common. In Denmark, concerns that higher tobacco prices would lead to an increase in cross-border trade from neighbouring countries to Denmark long prevented the raising of taxes [31].

## 2. Global, European and national determinants have influenced the adoption and implementation of preventive tobacco policies in the Nordic countries (see Figure 2)

### 2.1. Global and European regulations

All Nordic countries acceded to the WHO FCTC early. Norway was the first and has overall contributed to tobacco prevention being a high priority on the WHO’s agenda. In this, a significant role has been played by Gro Harlem Brundtland, former director-general of the WHO, who established the tobacco free initiative and initiated the WHO FCTC negotiations [32]. Overall, the Nordic countries have been actively involved in shaping and advancing global tobacco control policies. For instance, Finland was proactive in drafting resolutions calling for an international tobacco control treaty in 1995–1996 [32], and the Finnish public health strategy ‘’Health 2015’ in 2001 included a statement of the aim to achieve the WHO FCTC [33]. Once the treaty was established, Finland was one of the facilitators in the working group, with Sweden and Iceland among the partners, drafting the guidelines for the implementation of Article 13 [34]. Currently, the Finnish Institute for Health and Welfare hosts one of the WHO FCTC knowledge hubs. Norway has provided funding to the projects coordinated by the convention secretariat, including the FCTC2030 project [35], which supports parties in achieving the United Nations Sustainable Development Goals (SDGs) by accelerating the implementation of the WHO FCTC. In its international development strategy, Norway also aims at strengthening the implementation of the convention in low-income countries [36]. Denmark and Norway have utilised the international framework by engaging in a formal external country evaluation of WHO FCTC implementation to strengthen their national tobacco control [31, 37].

As members of the EU, Finland, Denmark and Sweden are obliged to transpose the Tobacco Products Directive (2014/40/EU) (TPD), the Tobacco Taxation Directive (2011/64/EU**)** (TTD) and the Tobacco Advertising Directive (2003/33/EC) (TAD) into their national legislation. As members of the European Economic Area (EEA), Iceland and Norway are obligated to implement all relevant EU directives, including most of the tobacco directives, but not the TTD. Also, lack of effective EU-level standards on tobacco taxation, as stated in the recent evaluation of the TTD [38], may have led to a variance in tobacco taxes between countries. A recent assessment of the TAD [39] highlights the need to regulate cross-border advertising and promotion particularly on the Internet and social media, yet the TAD focus on the traditional advertising and promotion channels partly explains the variance in regulation on TAPS on the online platforms.

Due to the TPD, all countries require health warnings on cigarette packages and limitations for nicotine in e-cigarettes, yet TPD has not harmonised all national regulations: Sweden (on EU accession) and Norway (on EEA accession) have negotiated exemptions on smokeless tobacco sales based on the historic availability of snus in their market. Also, the countries’ varying regulations on novel tobacco and nicotine products, and even regarding the core policies, may be partly explained by the shortcomings of the TPD (e.g. regulations on HTPs and nicotine pouches) [40]. TPD has also caused negative consequences or delayed progress as, for instance, Finland and Norway had to open their national market to nicotine-containing e-cigarettes after implementing TPD. Also, in Denmark, the ban on characterising flavours other than tobacco and menthol in other tobacco products than cigarettes and RYO, such as in chewing tobacco [41], will come into force only when the EU law stemming from the TPD allows this.

### 2.2. Sound national objectives, strategies and legislation for tobacco control

The Nordic countries demonstrate different histories in tobacco legislation. The first tobacco legislations date to the 1960s in Iceland (Alcoholic Beverages and Tobacco Trading Act no. 63/1969); the 1970s in Norway (Tobacco Control Act 14/1973), Finland (Tobacco Act 693/1976), and Sweden (Act Regulating Some of the Marketing of Tobacco Products 1978:764); and the 1990s in Denmark (Act on the Labelling of Tobacco Products and on the Tar Content of Cigarettes 426/1990).

Finland was the first Nordic country to publish a national tobacco control strategy in 1997, providing recommendations for stakeholders such as ministries, municipalities and schools to prevent smoking [42]. In 2001, the first national reduction target was set for youth smoking prevalence [33]. In 2010, as the first to do so in the world, Finland implemented the endgame objective of its Tobacco Act, aiming at a less than 5% tobacco use prevalence by 2040. The endgame goal was argued to illustrate the fundamental aim of tobacco control. It was also an important message to the tobacco industry and tobacco retailers regarding progressive restrictions on manufacture, distribution and supply [26]. In 2016, the goal was broadened to cover all non-medicinal nicotine products and moved the target forward to 2030.

In Norway, the first long-term strategy plan for tobacco control was adopted in 1999, and the most recent strategy is for 2019–2021 (Table I). Norway does not have a set endgame objective as Finland does, but in 2013 the Norwegian Tobacco Control Act objective was amended to include the goal of a tobacco-free society [43]. Denmark implemented the first national action plan in 2019, aiming comprehensively to stop and prevent children and adolescent smoking and nicotine addiction [41]. In Sweden, the government acknowledged and supported the NGO-led Smoke-Free Sweden 2025 in its strategy on alcohol, narcotics, doping and tobacco [44]. Iceland does not have an official tobacco control objective or strategy, but it has successfully implemented the Icelandic prevention model, which also targets smoking prevention [45, 46].

**Table I.** Tobacco control objectives and strategies in the Nordic countries in 2022.

Country	Denmark	Finland	Iceland	Norway	Sweden
National tobacco control strategies in force	National action plan against children and adolescent smoking [41]	Substance abuse and addiction strategy – common guidelines for 2030 [47]	No current strategy	National tobacco strategy 2019–2021 [48, 49]	ANDT strategy 2016–2020 [44] Proposal for the new ANDT strategy 2021–2025 [50]
Objectives of the current national tobacco control strategy	Prevent and stop children and adolescents from smoking and nicotine addiction	End the use of tobacco and nicotine products by the year 2030	No current objectives	Reduce the amount of people smoking daily below 10%, the use of snus among young people should not increase, and knowledge about the use of tobacco among pregnant women should increase	Reduce access to tobacco, decrease early first use of tobacco by young people, reduce tobacco use in adults and children (below 5% daily smokers), give better access to treatment, decrease tobacco-related morbidity and mortality

ANDT: Alcohol, narcotics, doping and tobacco.

### 2.3. Strong national structures for tobacco control

In the Nordic countries, the ministries of health have a leading role in tobacco control, and their commitment partly explains the rather comprehensive policies compared to many other European countries [7, 23]. Norway’s strong tobacco control history may be largely explained by its strong health ministry [37]. A governmental office for tobacco control (the National Council on Tobacco and Health) was already established in 1971 [51]. The WHO awarded the Finnish Ministry of Social Affairs and Health for its long-term commitment to tobacco control and its exemplary actions to protect young people from tobacco, especially e-cigarettes, on World No Tobacco Day in 2020 [52]. In Denmark, strong political commitment was not in place until recent years, which can be seen in Denmark’s relatively weak tobacco control history [31].

The strength of the health ministries in many Nordic countries has been supported by the establishment of tobacco control units, referring to a specialised agency or unit responsible for tobacco control. For instance, in Norway, the Norwegian Institute of Public Health (NIPH) works directly under the Ministry of Health and Care Services and produces knowledge on tobacco policy effectiveness and feasibility of implementation, and it thus guides the adoption and implementation of policies at the national level.

The financial resources for national tobacco control vary by country. The guidelines for implementing WHO FCTC Article 6 recommend that countries consider dedicating tobacco tax revenue to tobacco control programmes, such as those covering raising awareness, health promotion and disease prevention and cessation services [53]. However, none of the Nordic countries earmark a percentage of tobacco taxation income for the funding of any national plan or strategy on tobacco control. Nevertheless, in Iceland, 0.9% of all sold tobacco revenue is earmarked for tobacco control and tobacco prevention. The money has been distributed through a public health fund since 2011 [23, 54, 55]. Insufficient funding and resources have been highlighted as a barrier for sustaining progress in Norway [37], and for achieving the endgame goal by 2030 in Finland [56].

### 2.4. Active participation of civil society

NGOs have actively participated in formulating tobacco prevention in many Nordic countries. Strong NGOs exist in Sweden, Finland and Denmark, whereas in Norway their role has in recent years been minor [37]. Strong NGOs are often associated with governmental funding [57]. The NGOs have contributed to tobacco policy adoption by setting agendas, framing policies and building intersectoral collaboration. In Sweden, the NGOs led and formulated the objective for Smoke-Free Sweden 2025 [58], and have recently expressed their concerns on the stagnation in progress in national tobacco prevention [59]. In Denmark, the Danish partnership Smoke-Free Future anchored in the Danish Cancer Society initiated a collaboration in 2017 with the WHO regional office for Europe (WHO Europe) and the European Network for Smoking and Tobacco Prevention (ENSP) to drive progress in Danish tobacco control by engaging in a WHO FCTC capacity assessment [31]. In Finland, the intersectoral collaboration within the Tobacco-Free Finland network led by ASH Finland partly explains Finland’s favourable progress in tobacco control, especially during the past decade [52, 56]. Civil society’s input in keeping tobacco issues and the best solutions on policy agendas is crucial to maintain sustainable progress in tobacco prevention when the decision-makers’ interest may decrease after tobacco control objectives are achieved [56].

### 2.5. Preventing tobacco industry interference

The WHO FCTC Article 5.3 requires parties to protect their public health policies from the commercial and other vested interests of the tobacco industry [60], yet according to a recent comprehensive assessment, countries vary in the implementation of the measure [61]. Regarding the Nordic countries, so far only Norway has a national strategy in harmony with Article 5.3 [48, 49].

The tobacco industry influences all the Nordic countries, yet the presence appears to be most prominent in countries with their own tobacco manufacturing: snus and nicotine pouches are produced in Sweden, and snus, pipe tobacco, and cigars are made in Denmark. In Denmark, the tobacco industry’s active influence on policymakers and the public [62] has significantly contributed to Denmark’s slow progress in tobacco control in recent decades [31]. In Sweden, the snus industry has been active in lobbying local politicians and members of the European parliament, with invitations to events and seminars, and direct contacts to politicians. Snus is being presented as a product intertwined with Swedish cultural history, even though the growing and the production of tobacco for snus has occurred elsewhere for decades [63].

Overall, collaboration between the multinational tobacco companies, Nordic national manufacturer associations and local companies has delayed the implementation of smoke-free laws and health warnings on tobacco packages [64]. In Norway, Swedish Match aimed to delay the legislation on plain packaging by arguing that the Norwegian government was in breach of the free EEA trade rules and that the plain packaging of snus boxes was not in line with the health risks associated with snus. The court rejected Swedish Match’s claims, ruling that plain packaging was an internationally recommended and effective measure in line with the EEA [65].

### 2.6. Powerful public opinion

Public opinion is considered as a prerequisite for policy adherence and thus for enacting greater tobacco control [56]. A recent study [66] showed that public opinions on tobacco control differ by smoking status. Daily smokers viewed stricter tobacco control policies and workplace smoking bans more negatively and the availability of tobacco products more positively, as well as more often considered the present tobacco policy sufficient. Regardless of the smoking status, all showed positive attitudes towards the prevention of youth smoking [66]. With respect to this, tobacco policies are often framed to protect future generations from the harms of tobacco (i.e. the ‘child frame’). This was the case also in Denmark, where an increase in adolescent smoking fuelled widespread public pressure for political action that led to a comprehensive tobacco control strategy [41] and considerable improvements in tobacco control [31]. This further shows how public opinion is a powerful tool for facilitating agenda setting and policy adoption.

**Figure 2.**
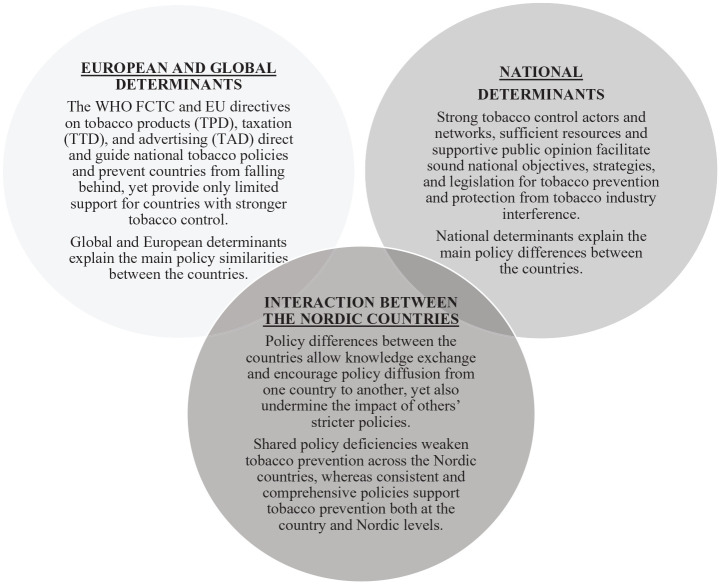
Summary of the global, European, Nordic and national determinants on the adoption and implementation of the preventive tobacco policies in the Nordic countries.

## Discussion

Our results show that the core preventive measures required by the WHO FCTC are rather comprehensively in place in the Nordic countries, and the countries have also implemented many of the advanced policies recommended in the treaty. However, individual weaknesses and shared deficiencies across the countries also exist that continue to undermine tobacco prevention. Our results inform the current tobacco control comparisons, such as the Tobacco Control Scale [7], by demonstrating the importance of considering the evolving tobacco control landscape when evaluating policy comprehensiveness.

The emergence of policies is a complex process determined by the interactions between actors holding power [11, 67]. Our results demonstrate how differences in the power, commitment and networking of the national tobacco control actors explain differences in countries’ tobacco policies. The key actors, namely the health ministry (strongest in Finland, Norway and Iceland), civil society (strongest in Sweden, Denmark and Finland) and public opinion (strongest in Denmark), have facilitated countries to implement many advanced policies among the first countries in the world: the endgame objective and flavour bans on e-cigarettes (Finland), outdoor smoking bans (Sweden), plain packaging (Norway), plain packaging on e-cigarettes (Denmark) and display bans (Iceland). In addition, if the current legislative proposal is passed, Iceland will become the first Nordic country prohibiting appealing flavours in nicotine pouches. Ensuring resources for these national actors and their coordinated collaboration in line with WHO FCTC Article 5.2. is important for sustainable progress in tobacco prevention, such as for strengthening the national strategies to provide protection from the tobacco industry’s interference. Currently, only Norway has strategies in line with WHO FCTC Article 5.3., yet the tobacco industry’s interference seems strongest in Sweden and Denmark.

Intersectoral collaboration is also emphasised in the health in all policies (HiAP) approach, which aims to consider health, wellbeing and equity in all policymaking, and thus to enforce health-promoting environments [68]. As HiAP can be excellently implemented in tobacco control, communicating the national tobacco prevention via HiAP could ensure commitment to tobacco prevention across the policymakers and thus facilitate the implementation of more comprehensive tobacco policies. Furthermore, HiAP could provide a valuable approach to build joint public health and tobacco control objectives and strategies across the Nordic countries that currently seem somewhat distinct from each other. Strengthening HiAP in global and European regulations in line with Europe’s Beating Cancer Plan [1] and its implementation roadmap [69] could encourage the better integration of HiAP at the national and Nordic levels.

The WHO FCTC and EU directives on tobacco products (TPD), taxation (TTD) and advertising (TAD) have harmonised tobacco policies in the Nordic countries in recent decades and ensured that the core preventive measures are in place, such as the 18-year age limit for sales, indoor smoking bans in public places and warning labels on tobacco packages. A recently published assessment on the WHO FCTC’s impact on tobacco control progress in 12 countries [70] supports our interpretations on the significance of the shared standards by indicating that the WHO FCTC had broadened political support for tobacco control, urged cross-sectoral collaboration, promoted the strong role of civil society and provided a comprehensive roadmap of legal obligations used by governments and courts to overcome the tobacco industry’s interference with the introduction of new policies [70]. However, despite the recent important decisions on the application of the provisions to novel products such as HTPs, more comprehensiveness is still needed, as countries are currently only invited to consider regulating e-cigarettes [24].

Despite the various benefits of EU directives, they also lack strength and provide only limited support, especially for countries with stronger tobacco control. This has led to policy differences and shared deficiencies in the Nordic countries, which are seen especially in the regulation on novel products and advertising in social media: the TPD does not extend to all novel and emerging tobacco products [40], the TTD lacks effective and consistent tax and price measures and regulation on novel nicotine products [38], and the TAD does not cover new global marketing channels such as social media [39]. Concerns over industry interference have resurfaced in the light of the TPD revisions, as a recent report reveals contacts between the tobacco industry, its allies and pro-vaping groups and the European Commission [71]. Strict compliance with WHO FCTC Article 5.3 should be enforced during the TPD revisions. In addition, countries should not be forced to hinder or delay their policy implementation due to TPD, which was seen in our results with a ban on domestic sales on novel products and a ban on flavours in smokeless tobacco products.

Nordic collaboration and participation in reinforcing the European regulations, resources for networking between the national tobacco control actors and national regulations to provide protection from the tobacco industry’s interference are needed to implement more comprehensive preventive tobacco policies in the Nordic countries. Potential strategies for facilitating the process are demonstrated in Table II. These strategies may also support implementation of other significant supply and demand reduction policies, such as the monitoring of tobacco use (WHO FCTC Article 20), cessation support (WHO FCTC Article 14) and preventing the illicit trade of tobacco (WHO FCTC Article 15).

**Table II.** Strategies to strengthen the preventive tobacco policies and facilitate their adoption and implementation.

Strengthening the tobacco policies	Strategies to facilitate policy adoption and implementation
**Extending regulations to all products** Stronger protection from the tobacco / nicotine industry to reduce the number or availability of tobacco and nicotine products on the markets. Further measures to control the supply of nicotine products entering the domestic and European markets. Extending the regulations to all tobacco and nicotine products. **High and consistent tax and price measures** Increasing taxes and prices on all tobacco and nicotine products. Ensuring that tax measures apply also to novel products. Ensuring a high minimum price for all products. **Extending prohibitions on TAPS to novel channels** Extending regulations on TAPS to also cover contemporary advertising channels, such as social media and packages of all products. Online sales should be banned as they inherently involve tobacco advertising and promotion. **Consistent implementation of advanced tobacco policies across the countries** Increasing implementation of the advanced measures recommended by the WHO FCTC, such as comprehensive outdoor smoking bans, plain packaging, flavour bans on all tobacco and nicotine products, bans on distance purchasing, and a minimum age of 20 or 21 years for sales.	**National** Developing national endgame objectives and strategies to prevent and reduce tobacco consumption, nicotine addiction, and exposure to tobacco smoke in line with the WHO FCTC and Europe’s beating cancer plan. Applying the HiAP approach and ‘child frame’ to strengthen societal and political support for tobacco prevention. Classification of all novel and emerging tobacco and nicotine products as tobacco products or other integration of these products into the national tobacco control regulations to prevent novel products from circumventing the regulations. Providing essential resources for sustainable progress, for instance, by earmarking money from tobacco taxes for prevention. Strengthening intersectoral collaboration and networking between the health ministry, tobacco control units, civil society, and other relevant actors, for instance, by allocating resources to coordinating efforts and co-operation. Developing national strategies in line with the WHO FCTC (Article 5.3) to protect tobacco control and public health policies from commercial and other vested interests of the tobacco industry. Countering tobacco industry interference. Establishing measures to limit interactions of public officials and civil servants with the tobacco industry and ensure the transparency of any interactions that occur. Governments should prohibit, or at least mandate the disclosure of, the tobacco industry’s donations of funds and in-kind contributions to political parties, trade unions or their foundations, and think tanks. The corporate social responsibility strategies of the tobacco industry should be de-normalized and prohibited. **Nordic** Collaboration to ensure consistent tobacco control objectives, strategies, and policies across the Nordic countries. Introducing and strengthening HiAP as a joint approach for decision making on tobacco prevention and public health. Activating networks for consultation and collaboration to ensure the diffusion and feasible implementation of policies from one country to another. Co-operation and coordinated efforts to limit legal and illicit cross-border advertising and trade as well as other phenomena that cause challenges to tobacco prevention across the countries. **Europe** Nordic countries’ active participation in developing the international and European regulations, policies, and policy guidelines (WHO FCTC, EU directives), for instance: 1. Investing in continuous production of scientific evidence to back up the global and European policy agreements. Strengthening the international requirements and guidance for policy implementation, especially the national strategies to provide protection from the tobacco industry in line with the WHO FCTC (Article 5.3.). 2. Advocating for extending TPD and TTD to cover all tobacco and nicotine products and strengthening the overall requirements of TTD. Supporting the revision of TAD to also cover contemporary advertising channels such as social media and packages of all tobacco and nicotine products.

EU: European Union; HiAP: health in all policies; TAPS: tobacco advertising, promotion and sponsorship; TPD: Tobacco Products Directive; TTD: Tobacco Taxation Directive; WHO FCTC: World Health Organization Framework Convention on Tobacco Control.

This is the first extensive preventive tobacco policy comparison in the Nordic countries that is based on the official documents on tobacco policy implementation. The policy comparisons illustrate the situation in 2020–2022 and may quickly change as new regulations are enacted. We were able to identify many potential determinants of policy adoption and implementation, yet certain aspects and data may be better represented than others, as the countries varied in terms of publicly available and easily accessible data. In this study, we assessed policy implementation with regard to national legislation and regulations, yet future studies should also focus on assessing the practical implementation and enforcement of these policies. In this process, attention should also be paid to the impact of countries on each other.

## Supplemental Material

sj-docx-1-sjp-10.1177_14034948221106867 – Supplemental material for Towards Tobacco-Free Generation: implementation of preventive tobacco policies in the Nordic countries Click here for additional data file. Supplemental material, sj-docx-1-sjp-10.1177_14034948221106867 for Towards Tobacco-Free Generation: implementation of preventive tobacco policies in the Nordic countries by Anu Linnansaari, Hanna Ollila, Charlotta Pisinger, Janne Scheffels, Jaana M. Kinnunen and Arja Rimpelä in Scandinavian Journal of Public Health

sj-docx-2-sjp-10.1177_14034948221106867 – Supplemental material for Towards Tobacco-Free Generation: implementation of preventive tobacco policies in the Nordic countries Click here for additional data file. Supplemental material, sj-docx-2-sjp-10.1177_14034948221106867 for Towards Tobacco-Free Generation: implementation of preventive tobacco policies in the Nordic countries by Anu Linnansaari, Hanna Ollila, Charlotta Pisinger, Janne Scheffels, Jaana M. Kinnunen and Arja Rimpelä in Scandinavian Journal of Public Health
