# Supplementary material for: Towards Tobacco-Free Generation: implementation of preventive tobacco policies in the Nordic countries
Source: Scand J Public Health. 2022 Jul 7;51(8):1108–21. doi: 10.1177/14034948221106867 (PMC10642214; doi:10.1177/14034948221106867)
Supplement: sj-docx-1-sjp-10.1177_14034948221106867 – Supplemental material for Towards Tobacco-Free Generation: implementation of preventive tobacco policies in the Nordic countries [file sj-docx-1-sjp-10.1177_14034948221106867.docx]

**Supplement 1. Assessed policies, their key indicators, and potential determinants for policy adoption and implementation.**

Table 1 presents our initial understanding on the different level determinants for policy adoption and implementation, groups the assessed preventive WHO FCTC policies under different Behavior Change Wheel (BCW) categories and specifies key indicators for each Article from the WHO FCTC Implementation database.

| **Determinants for policy adoption** | **Preventive WHO FCTC policies grouped under BCW categories** | **Key indicators for the Articles from the WHO FCTC Implementation Database*** |
| --- | --- | --- |
| **GLOBAL**  WHO FCTC (Articles 5.3, 8, 9, 11, 13)  United Nation’s (UN) Sustainable development goals (SDG) target 3a  and goal 3.4  **EUROPE**  European Union (EU) directives on tobacco products (TPD), taxation (TTD) and advertising (TAD)  **NORDIC**  Policy transfer (Cairney et al 2012, p. 23-44) consistency between the Nordic countries (WHO FCTC Articles 4  and 5)  Nordic contribution to international collaboration  (WHO FCTC Article 5 and 22)  **NATIONAL**  National strategies and objectives for tobacco control and prevention (WHO FCTC Article 5, Cairney et al 2012,  p. 23–44)  Tobacco legislation (WHO FCTC Article 5)  Tobacco control infrastructure, e.g., funding, role of health departments (WHO FCTC Article 5)  Societal factors, such as smoking prevalence in the country (Cairney et al 2012, p. 23–44)  Intersectoral collaboration between national tobacco control actors (WHO FCTC Articles 4 and 5, Cairney et al 2012, p. 23–44) and civil society participation (FCTC article 4, Cairney et al 2012, p. 23–44)  Protection from tobacco industry interference (WHO FCTC Article 5.3, Cairney et al 2012, p. 23–44) | **REGULATION (BCW)**  **Content of tobacco products (WHO FCTC Article 9)**  Reduce the attractiveness, addictiveness, and toxicity of tobacco products by adopting and implementing effective legislative, executive, and administrative or other measures for the testing and measuring as well as regulating the contents and emissions of tobacco products.  **Sales to and by minors (WHO FCTC Article 16)**  Prohibit the sales of tobacco products to or by persons at least under the age of 18 years, as well as limit the access of underage persons to tobacco products through not selling of tobacco products individually or in small packets and ensuring that tobacco vending machines are not accessible to minors or banned in total.  **COMMUNICATION AND MARKETING (BCW)**  **Packaging and warning labels (WHO FCTC Article 11)**  Ensure that tobacco products carry warnings covering an average of at least 50% but not less than 30% of the package with all appropriate  characteristics:  • Speciﬁc warnings are mandated and rotated  • Describe the harmful eﬀects of tobacco use on health  • Located on individual packs and any outside packaging used in retail sale  • Large, clear, visible, and legible  • Written in (all) principal language(s) of the country  • Including pictures or pictograms  **Advertising, promotion, and sponsorship (WHO FCTC Article 13)**  Undertake a comprehensive ban covering all types of direct or indirect advertising, promotion or sponsorship in different channels: national television and radio, local magazines and newspaper, millboards (and all other outdoor advertising), point-of-sale advertising, free distribution by mail or other means, promotional discounts, non-tobacco goods and services identiﬁed with brand names, brand name of non-tobacco goods and services used for tobacco products, appearance of tobacco brands or products in television and ﬁlms, sponsored events  **ENVIRONMENTAL / SOCIAL PLANNING (BCW)**  **Smoke-free policies (WHO FCTC Article 8)**  Provide protection from exposure to tobacco smoke in all public places, such as healthcare, governmental facilities, educational facilities, universities, other indoor offices and workplaces, hospitality venues (e.g., bars, restaurants, cafés), public transportation  **FISCAL MEASURES (BCW)**  **Price and tax measures** **(WHO FCTC Article 6)**  Ensure increase in sales prices of tobacco products by increasing taxes to comprise at least 75% of retail price of the most popular brands of cigarettes and prohibit or restrict sales of tax- and duty-free tobacco products.  **LEGISLATION (BCW)**  WHO FCTC Articles 6, 8, 9, 11, 13, 16 | **Article 9:**  **Core policies:**  C231 – Testing and measuring the contents of tobacco products  C232 – Testing and measuring the emissions of tobacco products  C233 – Regulating the contents of tobacco products  C234 – Regulating the emissions of tobacco products  **Article 16:**  **Core policies:**  C321 – Sales of tobacco products to minors prohibited  C321a – Minimum legal age for sale/purchase of tobacco products**  C322 – Clear and prominent indicator required  C323 – Required that sellers request for evidence of having reached full legal age  C324 – Ban of sale of tobacco in any directly accessible manner  C325 – Manufacture and sale of any objects in the form of tobacco products prohibited  C327 – Tobacco vending machines not accessible to minors**  C3281 – Distribution of free tobacco products to the public prohibited  C3282 – Distribution of free tobacco products to minors prohibited  C3210 – Penalties against sellers provided  C3211 – Sale of tobacco products by minors prohibited  **Advanced policies:**  C326 – Sale of tobacco products from vending machines prohibited  C329 – Sale of cigarettes individually or in small packets prohibited  **Article 11:**  **Core policies:**  C251 - Packaging of tobacco products do not carry advertisement or promotion  C252 - Misleading descriptors banned  C253 - Health warnings required  C254 - Health warnings approved by the competent national authority  C255 - Rotated health warnings  C256 - Large, clear, visible and legible health warnings  C257 - Minimum requirements of warnings mandated by law  C258 - Health warnings occupying no less than 30% of the principal display area  C25131 – Packaging contains information of constituents of tobacco products  C25132 – Packaging contains information of emissions of tobacco products  C2514 - Warning required in the principal language(s) of the country  **Advanced policies:**  C259 - Health warnings occupying 50% or more of the principal display area  C2510 - Health warnings in the form of pictures or pictograms  **Article 13:**  **Core policies:**  C271 - Comprehensive ban on all tobacco advertising, promotion and sponsorship****  C2729 - Ban covering cross-border advertising originating from the country  **Advanced policies:**  C2721 - Ban on display of tobacco products at points of sales  C2722 - Ban covering the domestic internet  C2723 - Ban covering the global internet  C2724 - Ban covering brand stretching and/or sharing  C2725 - Ban covering product placement  C2726 - Ban covering the depiction/use of tobacco in entertainment media  C2727 - Ban covering tobacco sponsorship of international events, activities and/or participants therein  C2728 - Ban covering corporate social responsibility  C27210 - Ban covering cross-border advertising entering the country  **Article 8:**  **Core policies:**  C221 - Tobacco smoking banned in all public places****  C226a1 - Comprehensiveness of protection in government buildings  C226a2 - Comprehensiveness of protection in health-care facilities  C226a3 - Comprehensiveness of protection in educational facilities  C226a4 - Comprehensiveness of protection in universities  C226a5 - Comprehensiveness of protection in private workplaces  C226b1 - Comprehensiveness of protection in airplanes  C226b2 - Comprehensiveness of protection in trains  C226b3 - Comprehensiveness of protection in ferries  C226b4 - Comprehensiveness of protection in ground public transport  C226b5 - Comprehensiveness of protection in motor vehicles used for work (taxis, ambulances, delivery vehicles)  C226c1 - Comprehensiveness of protection in cultural facilities  C226c2 - Comprehensiveness of protection in shopping malls  C226c3 - Comprehensiveness of protection in pubs and bars  C226c4 - Comprehensiveness of protection in nightclubs  C226c5 - Comprehensiveness of protection in restaurants  **Advanced policies:**  C226b6 - Comprehensiveness of protection in private vehicles  **Article 6:**  **Core policies:**  C211 - Tax policies to reduce tobacco consumption**  **Advanced policies:**  B81 - Proportion of the retail price consisting of taxes***  B85 - Tobacco tax earmarking** |
| *****The division of the measures to core and advanced policies for this article has been conducted by HO by the strength of the language in the Convention. The core policies include measures required in the treaty with language indicating that Parties shall adopt or take other effective actions. The advanced policies include recommended measures in the treaty or in its implementation guidelines with language indicating that Parties shall endeavor to, should or may take actions, or where actions are listed after “as appropriate”, or where Parties have the option to restrict instead of taking effective actions.  ** Not included in the calculation, covered only for the text.  *** For the proportion of the total tax rate, most recent information from the WHO Global Tobacco Epidemic 2021 -report was utilized. | | |
