# Supplementary material for: Towards Tobacco-Free Generation: implementation of preventive tobacco policies in the Nordic countries
Source: Scand J Public Health. 2022 Jul 7;51(8):1108–21. doi: 10.1177/14034948221106867 (PMC10642214; doi:10.1177/14034948221106867)
Supplement: sj-docx-2-sjp-10.1177_14034948221106867 – Supplemental material for Towards Tobacco-Free Generation: implementation of preventive tobacco policies in the Nordic countries [file sj-docx-2-sjp-10.1177_14034948221106867.docx]

**Supplement 2. Comparisons of the preventive tobacco policies in the Nordic countries.**

Table 1. Comparison of the selected preventive WHO FCTC policies in the Nordic countries.

| **WHO FCTC** | **Reported status of implementation of the key indicators in the WHO FCTC Implementation Database** | **Key similarities and differences in tobacco policies** | **References** |
| --- | --- | --- | --- |
| ***Sales to and by minors (Article 16)*** | Number of measures implemented to prevent youth access and sales of products with special appeal to minors:  Iceland 11,  Finland 10,  Norway 10,  Sweden 8,  Denmark 7 | In all the Nordic countries, tobacco products can only be sold to people over 18 years. However, Denmark implemented age control measures later than the other countries, as sales to minors were prohibited in 2004 with an age limit of 16 years, which was later raised to 18 in 2008. The same age limit is applied to e-cigarettes in all countries. Norway and Denmark apply it to nicotine pouches, and Swedish and Icelandic governments have recently proposed it. In Finland nicotine pouches require a medicinal sales permit and are therefore subject to 18 years age limit.  Finland and Iceland prohibit the sale of tobacco products from self-service vending machines. In Norway and Denmark, neutrally designed machines can be used for delivering the product but not the sale itself, which must take place at a sales counter with age verification. In Norway, the delivery machine must in addition be placed near the cashier. Sweden allows selling tobacco products from vending machines but does not allow minors to access them. | Acts in Table 2.  Statens offentliga utredningar. Hårdare regler för nya nikotinprodukter. Report: betänkande av utredningen on översyn av vissa frågor på tobaksområdet. Stockholm, Sweden: SOU 2021:22.  The Danish government. 2019. National action plan against children and adolescent smoking. Report, Denmark, 2019.  WHO FCTC. WHO FCTC Implementation database and country reports. *Online referencing*, https://untobaccocontrol.org/impldb/2020 (2020, accessed 9 March 2022).  WHO FCTC. Best practices on implementation of the tobacco  advertising and display ban at point of sale (Article 13 of the WHO FCTC). A four-country study: Ireland, Norway, Finland and the United Kingdom. Report, 2016.  WHO. Capacity Assessment on the Implementation of Effective Tobacco Control Policies in Denmark. Report, Copenhagen, Denmark, 2018. |
| ***Price and tax measures (Article 6)*** | Percentage contribution of all tobacco taxes to the retail price of a pack of 20 of the most popular brands of cigarettes:  Finland 88%  Denmark 78%  Sweden 69%  Norway 62%  Iceland 55%  (WHO 2021a) | All Nordic the countries implement tax policies on tobacco products, but by 2021 only Finland and Denmark have met the WHO recommendation of a minimum of 75% tax share of the retail price of tobacco. In recent years, Denmark has increased the tax considerably, whereas Finland has increased taxes in small steps regularly since 2009. Both Finland and Denmark are now among the countries with the highest total tax in the EU. Smokeless tobacco is subject to taxation in Iceland (with a total tax of 62%), Norway (64%), Sweden (total tax rate not available), and Denmark (total tax rate not available).  The price of tobacco and nicotine products but also the consumers’ income are two major determinants of product demand. So, the affordability depends on the price relative to consumers’ income. Altogether, cigarettes became less affordable between 2010 and 2018 in Finland, Sweden, and Norway, but from 2018 to 2020 in all countries, where the change was the highest in Denmark and Finland and the least in Sweden and Iceland.  Most countries have inconsistent tax policies with traditional and novel products. Finland, Norway, and Sweden have excise tax for nicotine-containing liquids and nicotine-free liquids intended for vaporization. Denmark will introduce a tax on nicotine-containing e-liquids in 2022 and will furthermore introduce a tax on nicotine products in 2022. Heated tobacco products (HTPs) are sold in Denmark and Sweden, with the total tax being 43% in Sweden and 31% in Denmark. Although HTPs are not sold in Finland, they are already subject to taxation. | Acts in Table 2.  Nargis N & Stoklosa M. Price, Income, and Affordability as the Determinants of Tobacco Consumption: A Practitioner’s Guide to Tobacco Taxation*.* Nicotine & Tobacco Research 2021; 23(1), 40–47.  Retsinformation. Lov nr. 1182 af 8. juni 2021. Legislation, Denmark, 2021.  Skatteetaten. Avgift på tobakksvarer. Report, Norway, 2022.  WBG Global Tobacco Control Program Team. E-cigarettes: Use and Taxation. Report of World Bank Group, 2019.  WHO FCTC. Guidelines for implementation of Article 6: Price and tax measures to reduce the demand for tobacco. Report, Geneva, Switzerland, 2013.  WHO FCTC. WHO FCTC Implementation database and country reports. *Online referencing*, https://untobaccocontrol.org/impldb/2020 (2020, accessed 9 March 2022).  WHO. WHO report on the global tobacco epidemic 2021: Addressing new and emerging products. Web annexes: WHO Global Tobacco Control Policy Data. Report, Geneva, World Health Organization, 2021a.  WHO. WHO technical manual on tobacco tax policy and administration. Geneva, 2021. |
| ***Protection from exposure to tobacco smoke (Article 8)*** | Number of complete and partial smoking bans in required/ recommended settings:  Norway  15 complete    Iceland  11 complete,  2 partial  Denmark  4 complete,  11 partial  Sweden  3 complete,  12 partial  Finland  2 complete,  14 partial | Some variations may exist by school types, but in general, all the Nordic countries have implemented bans on cigarettes and e-cigarettes on the indoor and outdoor premises of schools, but Sweden has not prohibited smoking rooms for staff. In Finland, the use of products is prohibited for everyone on all school premises at all times. Norway prohibits tobacco and nicotine use completely for all in elementary schools and kindergartens, indoors and outdoors, and during school hours for pupils regardless of where they are. Tobacco- and nicotine-free school hours were implemented in Denmark in 2021 and the concept is included in the proposal for the new ANDTS strategy in Sweden, which, however, has not yet been approved by the government. Bans on the use of smokeless tobacco products exist in educational facilities in all countries except Sweden.  Regarding other public places, all Nordic countries have implemented smoking bans in indoor public places, workplaces, and public transport. However, exemptions exist, as smoking rooms with certain demands are sometimes allowed, especially in restaurants and bars in all countries except in Norway. In Denmark, there are several exemptions, for example, smoking can be allowed in bars if they fulfil several criteria, such as being smaller than 40m^2^. There are also exceptions for company cars. Finland prohibits smoking in private vehicles in the presence of adolescents under the age of 15. This regulation has recently been proposed by the government also in Norway.  Article 8 of the WHO FCTC also calls for the implementation of smoking bans in outdoor or quasi-outdoor public places, where appropriate. Sweden has been most progressive in implementing smoking bans to outdoor public places: smoking is prohibited on school grounds, areas outside childcare facilities, public playgrounds, terraces of cafés and restaurants, outdoor areas of public transport such as bus stops and train stations, and entrances to establishments, public venues, and other spaces to which the public has access. Also, Norway prohibits smoking outside the entrance to health institutions and public buildings. Finland has implemented smoking bans in audience areas at outdoor public events and recently extended the ban also to public playgrounds (also snus) and public beaches. Housing corporations in Finland may request the municipality to impose a smoking ban on the balconies or other areas of individual apartments. Similar regulation concerning outdoor playgrounds, bus stops, and sport venues has recently been proposed by the government also in Norway.  Countries differ in whether they have applied the smoking bans also to novel products. Smoking bans are applied to the use of e-cigarettes in all countries. HTPs are sold in Sweden, where smoking bans are also applied to them. Denmark is currently in the process of deciding which provisions to apply to the products. Also in Finland, where HTPs are not sold, the smoking bans are extended to HTPs. | Acts in Table 2.  Finlex. 2021. HE 141/2021 The government’s proposal to Parliament to amend the Tobacco Act. Legislative proposal, Finland, 2021. Ministry of Social Affairs and Health. Tobacco Act tightened to remove logos from packaging of tobacco products and electronic cigarettes. Press release, Finland, 2022. Norwegian government. Høring av endringer i tobakksskadeloven. Legislative proposal, Norway, 2021.  Regerinskansliet. En förnyad strategi för politiken avseende alkohol, narkotika, dopning, tobak och nikotin samt spel om pengar 2021–2025 Prop. 2020/21:132. Report, Sweden, 2021.  The Danish government. 2019. National action plan against children and adolescent smoking. Report, Denmark, 2019.  WHO FCTC. WHO FCTC Implementation database and country reports. *Online referencing*, https://untobaccocontrol.org/impldb/2020 (2020, accessed 9 March 2022). |
| ***Regulation of the contents of tobacco products (Article 9)*** | Number of measures to test and regulate the contents and emissions of tobacco products:  Denmark 4,  Finland 4,  Sweden 4,  Iceland 3,  Norway 1 | In line with the guidelines for implementing Article 9, there is a ban on flavours for cigarettes and roll-your-owns (RYO) in all Nordic countries, except in Norway and Iceland. Such a ban is however expected also in Norway and Iceland in 2022 as part of their implementation of the EU Tobacco Products Directive 2014/40/EU.  Finland prohibits other than tobacco flavours in e-cigarettes and nicotine-containing and nicotine-free e-liquids intended for vaporization. Denmark has banned other than tobacco or menthol flavours in electronic cigarettes and refill containers with and without nicotine. The Norwegian, Icelandic and Swedish governments have recently proposed flavour bans on e-cigarettes. Bans on flavours in smokeless products are not currently in place in any country selling smokeless tobacco products. Denmark has announced the intention to ban characterizing flavours other than tobacco and menthol in other tobacco products than cigarettes and RYO, for example chewing tobacco (however, excluding pipe tobacco and cigars), but the ban will come into force when the EU law stemming from the Tobacco Products Directive allows this. The Icelandic government has recently suggested a ban on appealing flavours in nicotine products, such as nicotine pouches. If the legislation is passed, Iceland will become the first Nordic country prohibiting appealing flavours in nicotine pouches. Smokeless tobacco products and nicotine pouches do not have regulations on nicotine content. | Acts in Table 2.  Ministry of Health. Nicotine products. Legislative proposal, Iceland, 2022.  Norwegian government. Høring av endringer i tobakksskadeloven. Legislative proposal, Norway, 2021.  Norwegian government. Nytt tobakksdirektiv innlemmes i EØS-avtalen. News report, Norway, 2022.  Statens offentliga utredningar. Hårdare regler för nya nikotinprodukter. Report: betänkande av utredningen on översyn av vissa frågor på tobaksområdet. Stockholm, Sweden: SOU 2021:22.  The Danish government. 2019. National action plan against children and adolescent smoking. Report, Denmark, 2019.  WHO FCTC. WHO FCTC Implementation database and country reports. *Online referencing*, https://untobaccocontrol.org/impldb/2020 (2020, accessed 9 March 2022). |
| ***Packaging and labelling of tobacco products (Article 11)*** | Number or required/recommended elements in health warnings:  Norway 11,  Denmark 11,  Finland 11,  Sweden 10,  Iceland 9 | All Nordic countries demand clear and visible health warnings on the packaging of all tobacco products. Iceland (implemented in 1969–1971, 1985) and Norway (implemented in 1973) were among the first in the world to require health warnings. Pictorial warnings were first implemented in Iceland in 1985. Norway introduced pictorial warnings for smoking tobacco in 2009, and Norway and Iceland will introduce the new and larger pictorial warnings when the TPD is fully implemented in 2022. Pictorial warnings are not required for snus in Sweden and Norway, nor for chewing tobacco in Sweden, Norway, Denmark, and Iceland. However, Norway will introduce an additional health warning on oral tobacco relating to harms to the foetus in 2022. Health warnings on e-cigarettes are in place in all Nordic countries. HTPs are subjected to a health warning in Denmark and Sweden. Health warnings for nicotine pouches are required in Denmark and Norway and are included in the latest legislative proposal in Iceland.  In 2017, Norway was the first Nordic country to enact plain packaging, as recommended in the Guidelines for implementing Article 11. It covers cigarettes, roll-your-own, and snus. A proposal to also include e-cigarettes was sent to public consultation in 2021. Additionally, in Denmark, all tobacco products except cigars and pipe tobacco will be required to be in plain packaging. Plain packaging will also apply to e-cigarettes, refill containers, and tobacco for HTPs but not to oral nicotine pouches. Finland will require plain packaging for all tobacco products. | Acts in Table 2.  Finlex. 2021. HE 141/2021 The government’s proposal to Parliament to amend the Tobacco Act. Legislative proposal, Finland, 2021. Folkhälsomyndigheten. Labelling and packaging of tobacco products. Report, Sweden, 2020. Hiilamo H, Crosbie E, Glantz SA  The evolution of health warning labels on cigarette packs: the role of precedents, and tobacco industry strategies to block diffusion. *Tobacco Control* 2014; 23: e2. Ministry of Social Affairs and Health. Tobacco Act tightened to remove logos from packaging of tobacco products and electronic cigarettes. Press release, Finland, 2022. Norwegian government. Høring av endringer i tobakksskadeloven. Legislative proposal, Norway, 2021. WHO FCTC. WHO FCTC Implementation database and country reports. *Online referencing*, https://untobaccocontrol.org/impldb/2020 (2020, accessed 9 March 2022). |
| ***Tobacco advertising, promotion, and sponsorship (TAPS) (Article 13)*** | Number of types of tobacco advertising, promotion, and sponsorship bans:  Norway 9,  Finland 9,  Sweden 7,  Iceland 7,  Denmark 5 | While TAPS is mostly prohibited in all Nordic countries, some exemptions exist. The advertising ban in TAPS is not reported to cover the global Internet in Sweden, Iceland, or Denmark. However, a clear definition on the means integrated in the global Internet is not provided in the WHO FCTC implementation guidelines for Article 13, which may have led to different interpretations of the measure. Direct advertising on the Internet is banned in all the Nordic countries. Iceland is the only country not prohibiting cross-border advertising from entering the country, and Iceland and Denmark have not prohibited cross-border advertising originating from the country. Finland is the only country reporting to impose penalties for offences. Finland is the only Nordic country prohibiting the purchase of all tobacco products via distance communication, such as the Internet or email. Iceland is the only Nordic country banning the depiction and use of tobacco products in entertainment media.  Iceland was the first country in the world to enact a point-of-sale display ban for tobacco products in 2001, a measure recommended in the guidelines for the implementation of Article 13. All other Nordic countries except Sweden have since implemented the policy. In Denmark, Norway, and Iceland, which allow online sales, the display ban covers also online stores, meaning that images of the products may not be shown to the customer. In Denmark, images of pipes are not included in the ban. In Norway, online pictorial displays and promotional material or information is prohibited in locally based online shops. Finland, Norway, and Denmark apply a display ban also to e-cigarettes. In Denmark, the display ban is also extended to HPTs and nicotine pouches.  Prohibition on TAPS is fully extended to e-cigarettes in Finland, Denmark, Iceland, and Norway, covering all the same direct and indirect forms of TAPS as for tobacco products, and partially also in Sweden. Swedish government has recently proposed stricter regulations on TAPS regarding tobacco-free nicotine products. Regulation on TAPS is extended to HTPs in Sweden and Denmark, and also to nicotine pouches in Denmark. In Finland, the display ban concerning HTP devices is included in the latest legislative proposal. Finland is the only Nordic country prohibiting the purchase of all tobacco products (i.e. tobacco products, e-cigarettes, and nicotine containing e-liquids) via distance communication, such as the Internet or email. | Acts in Table 2.  Finlex. 2021. HE 141/2021 The government’s proposal to Parliament to amend the Tobacco Act. Legislative proposal, Finland, 2021.  Statens offentliga utredningar. Hårdare regler för nya nikotinprodukter. Report: betänkande av utredningen on översyn av vissa frågor på tobaksområdet. Stockholm, Sweden: SOU 2021:22.  WHO FCTC. Best practices on implementation of the tobacco  advertising and display ban at point of sale (Article 13 of the WHO FCTC). A four-country study: Ireland, Norway, Finland and the United Kingdom. Report, 2016.  WHO FCTC. WHO FCTC Implementation database and country reports. *Online referencing*, https://untobaccocontrol.org/impldb/2020 (2020, accessed 9 March 2022).  WHO. WHO report on the global tobacco epidemic 2021: Addressing new and emerging products. Web annexes: WHO Global Tobacco Control Policy Data. Report, Geneva, World Health Organization, 2021a. |

Table 2. The main acts regulating tobacco control in the Nordic countries in 3/2022.

| **Country** | **Denmark** | **Finland** | **Iceland** | **Norway** | **Sweden** |
| --- | --- | --- | --- | --- | --- |
| **Main acts regulating tobacco and nicotine products** | The Act on tobacco products, etc. (LBK no. 1489 from 18/06/2021)  Act on electronic cigarettes and refill containers for electronic cigarettes (LBK no 1876 form 20/09/2021) | Tobacco Act (549/2016) | Tobacco Control Act (6/2002)  The Act on electronic cigarettes and refill containers for electronic cigarettes (87/2018) | Tobacco Control Act (14/1973)  The Act amending the Tobacco Control Act (5/2017)  Regulation on approval scheme for new tobacco and nicotine products, in force from 1 July 2021 (2021-06-17, 2131) | The Act on tobacco and similar products (2018:2088)  Decree on tobacco and similar products (2019:223) |
| **References** | <https://www.retsinformation.dk/> | <https://www.finlex.fi>  <https://www.finlex.fi/en/laki/kaannokset/2016/en20160549_20161374.pdf> | <https://www.government.is/media/velferdarraduneyti-media/media/acrobat-enskar_sidur/Tobacco-Control-Act-as-amended.pdf>  <https://samradsgatt.island.is/oll-mal/$Cases/Details/?id=2866&uid=680b0b97-ff63-eb11-9b9f-005056bcce7e>  <https://www.government.is/news/article/2018/09/20/New-Legislation-on-E-cigarettes-Will-Take-Effect-on-1-March-2019/> | <https://www.regjeringen.no/en/search/id86008/?term=tobacco+control+act>  <https://lovdata.no/dokument/SF/forskrift/2003-02-06-141?q=forskrift%20om%20merking%20av%20tobakksvarer>  <https://lovdata.no/dokument/SF/forskrift/1995-12-15-989>  <https://lovdata.no/pro/#document/SF/forskrift/2021-06-17-2131> | <https://www.riksdagen.se/sv/dokument-lagar/dokument/svensk-forfattningssamling/lag-20182088-om-tobak-och-liknande-produkter_sfs-2018-2088>  <https://www.riksdagen.se/sv/dokument-lagar/dokument/svensk-forfattningssamling/forordning-2019223-om-tobak-och-liknande_sfs-2019-223>  <https://www.riksdagen.se/sv/dokument-lagar/dokument/statens-offentliga-utredningar/hardare-regler-for-nya-nikotinprodukter_H9B322> |
